# GeneChaser: Identifying all biological and clinical conditions in which genes of interest are differentially expressed

**DOI:** 10.1186/1471-2105-9-548

**Published:** 2008-12-18

**Authors:** Rong Chen, Rohan Mallelwar, Ajit Thosar, Shivkumar Venkatasubrahmanyam, Atul J Butte

**Affiliations:** 1 Stanford Center for Biomedical Informatics Research, Department of Medicine, Stanford University School of Medicine, 251 Campus Drive, Stanford, CA 94305, USA; 2 Department of Pediatrics, Stanford University School of Medicine, 251 Campus Drive, Stanford, CA 94305, USA; 3 Lucile Packard Children's Hospital, 725 Welch Road, Palo Alto, CA 94304, USA; 4 Optra Systems Pvt. Ltd, 1, "Dnyanesh", CTS No. 1179/3, Modern College Road, Shivajinagar, Pune, 411 005, India

## Abstract

**Background:**

The amount of gene expression data in the public repositories, such as NCBI Gene Expression Omnibus (GEO) has grown exponentially, and provides a gold mine for bioinformaticians, but has not been easily accessible by biologists and clinicians.

**Results:**

We developed an automated approach to annotate and analyze all GEO data sets, including 1,515 GEO data sets from 231 microarray types across 42 species, and performed 12,658 group versus group comparisons of 24 GEO-specified types. We then built GeneChaser, a web server that enables biologists and clinicians without bioinformatics skills to easily identify biological and clinical conditions in which a gene or set of genes was differentially expressed. GeneChaser displays these conditions in graphs, gives statistical comparisons, allows sort/filter functions and provides access to the original studies.

We performed a *single gene search *for *Nanog *and a *multiple gene search *for *Nanog*, *Oct4*, *Sox2 *and *LIN28*, confirmed their roles in embryonic stem cell development, identified several drugs that regulate their expression, and suggested their potential roles in sex determination, abnormal sperm morphology, malaria infection, and cancer.

**Conclusion:**

We demonstrated that GeneChaser is a powerful tool to elucidate information on function, transcriptional regulation, drug-response and clinical implications for genes of interest.

## Background

The amount of gene expression data in the public repositories, such as NCBI Gene Expression Omnibus (GEO) [1] has grown exponentially, and provides a gold mine for bioinformaticians, but has not been easily accessible by biologists and clinicians. Microarrays measure the expression of thousands of genes simultaneously, and have revolutionized basic and clinical research by enabling the unbiased discovery of sets of genes whose expression is characteristic of a given cell type, treatment or disease state. Since the advent of this technology more than a decade ago, we have accumulated the expression levels of essentially all genes in the genomes for more than 100 species, across many tested biological conditions, such as diseases, gene-knockouts, drug responses, genotype variations, and more. There is still a need for a bioinformatics resource to analyze these data and enable biologists and clinicians to easily identify all experimental conditions in which a given gene or set of genes is differentially expressed, which would be very useful in elucidating the biological functions, transcriptional regulation, drug-response and clinical implication of those genes.

Before 2001, microarray data could only be retrieved individually from lab web sites. GEO was built in 2001 as a first generation centralized repository of microarray data to facilitate the submission, storage, and retrieval. A European counterpart, ArrayExpress [2], was later built by European Bioinformatics Institute. These databases have been tremendously successful, as evidenced by the exponential increase of deposited data. As of this writing, GEO contained 256,691 microarray samples from 9,988 experiments across over 100 species using 5,134 types of microarrays, summing over nine billion expression measurements. Furthermore, the count of microarrays has been doubling or tripling every year. GEO now contains expression measurements for all human genes in 2,394 conditions, including 139 diseases. However, biologists lacking bioinformatics skills have difficulties using and interpreting the data in these repositories. As a result, a second generation of microarray databases was designed to select and analyze subsets of publicly-available microarrays and display these using a user friendly interface. For example, GNF SymAtlas [3] contains expression profiles for most protein-coding genes in 79 human and 61 mouse tissues. The Connectivity Map [4] identifies expression profiles of different cell lines after perturbation by a catalog of small molecule drugs. L2L [5] manually collected 357 lists of genes that were differentially expressed under different stimuli enabling the comparison with any list of genes of interest to identify their underlying patterns. Oncomine [6] contains the manually collected and analyzed expression profiles of 40 cancer types from 25,447 microarray samples in 360 experiments. NextBio [7] manually analyzed some data from GEO, ArrayExpress, and Stanford Microarray Database and delivered expression profiles for a larger number of tissues, diseases, and treatments. The creators of these databases performed a handful of experiments or manually curated a subset of experiments from public repositories, and delivered the results for some types of conditions, such as tissue expression in SymAtlas, drug perturbation in connectivity map and cancer profiling in Oncomine. Nextbio processed a large number of experiments from multiple repositories, but the data sets were still manually curated for only three types of experimental conditions, such as tissue type, disease and treatments. Although very useful, these databases are relatively restricted in their applications, and there is still a need for a resource to quickly process whole data sets from repositories for all types of conditions.

We have previously developed a fully automated tool AILUN [8] that reannotates the complete data from GEO for all types of arrays across all species. This paper describes a web server, GENE CHAnge browSER (GeneChaser[9]) that automatically reannotates and analyzes all GEO data sets (GDS), performs all group versus group comparisons, identifies all experimental conditions where the expression levels of a gene or set of genes is significantly changed, and displays them graphically with statistical comparisons and sort/filter functions. It also provides access to the raw data in the original studies. We provide examples that show how well-known genes involved in stem cell pluripotency can be used as search criteria, yielding previously known and unknown conditions that alter these genes.

## Results

### Microarray data analysis

We used AILUN [8] to parse and reannotate 1,515 GEO data sets (GDS) spanning 231 microarray platforms (GPL) and 42 species with the latest probe-gene annotations. We performed all group versus group comparisons within each GDS. For example, GDS2654, a study of age-related neurological senescence in mice, was annotated with 4 experimental variables, including disease state, strain, tissue, and age. There were 2 groups in the disease state, resulting in 1 comparison: accelerated aging versus normal. There were 3 groups in strain, resulting in 3 comparisons: SAMP1 versus SAMP8, SAMP1 versus SAMP10, and SAMP8 versus SAMP10. Similarly, we performed 1 tissue comparison, hippocampus versus retina, and 1 age comparison, 3 month versus 16 month. Comparisons were constrained on groups with 3 or more samples. In total, we performed 12,658 comparisons across 1,515 GDSs, involving 31,602 total microarrays.

For every comparison, we recorded all differentially expressed genes and their corresponding q-values with a false discovery threshold of 0.2 (q ≤ 0.2) using Significance Analysis of Microarray (SAM) [10] in the siggenes package of Bioconductor. In 5,457 of 12,658 comparisons, at least one gene was differentially expressed at a q-value ≤ 0.05. On average, each comparison contained 1,857 differentially expressed genes and each gene was differentially expressed in 54 comparisons and 17 GDSs. A lot of diseases have been studied in multiple species. To identify differentially expressed conditions across species, we considered groups of orthologous genes as defined in Entrez Homologene [11]. Of 45,205 ortholog groups in Homologene, the expression of 37,998 groups was measured in one or more comparisons, of which 35,572 groups (comprised of 199,678 genes from 20 species) showed differential expression in at least one comparison. Each Homologene group was on average differentially expressed in 233 comparisons and 71 GDSs.

### GeneChaser

We then developed GeneChaser, a web server that identifies and visualizes comparisons in which a gene or set of genes was significantly differentially expressed. We implemented two search functions, *single gene search *and *multiple gene search*. *Single gene search *takes as input any gene identifier, such as a gene symbol or Swissprot ID, and returns comparisons relevant to the differential expression of that gene and optionally its orthologs. The underlying universal gene identifier table [8] contains 83 million identifiers for 3.6 million genes from 4578 species, enabling us to recognize most gene identifiers, even a university clone ID or IMAGE clone ID.

### Single gene search

We performed a *single gene search *in all species for Nanog, a key transcription factor necessary for pluripotency in embryonic stem (ES) cells [12]. Nanog, or one of its orthologs, was measured in 5,706 comparisons and was differentially expressed in 217 of them (q ≤ 0.05, fold > 2) (Additional File 1, [13]). The most significant change in Nanog expression was 1000-fold upregulation in ES cells compared to hematopoietic stem cells (q = 0.006, GDS2718). Within the top 15 studies where Nanog was up-regulated by more than 25 fold, all except two tissue comparisons were studies of preimplantation embryonic developments in different strains of mice. These studies reveal continuously increased expression of Nanog as an organism progressed from being an oocyte to having 1, 2, and 8 cells, and finally to the blastocyst stage. The increased expression is the most significant at 8-cell stage. This clearly confirms a role for Nanog in ES cell development, consistent with the current understanding of embryonic development as well as recent experiments with induced pluripotent cells [14].

To better understand the impact of different factors on Nanog expression, we filtered the above comparisons by comparison types. Currently, there are 24 GEO-specified comparison types, including disease state, genotype/variation, strain, infection, development stage, age, time, agent, dose, tissue, cell type, cell line, metabolism, stress, growth protocol, protocol, gender, individual, isolate, shock, species, specimen, temperature, and others.

**Figure 1:**
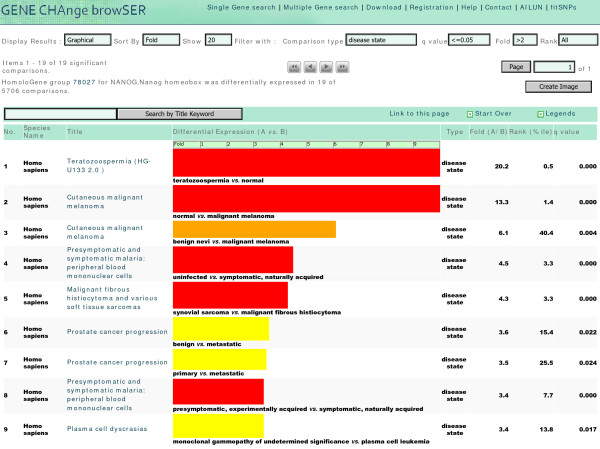
**Top nine disease state comparisons showing differential expression of Nanog**. A *single gene search *for Nanog showed that it was measured in 5706 experimental conditions, and differentially expressed in 19 disease states in human studies (fold > 2, q ≤ 0.05). Bar colors represent q-values, length represents fold change, and width represents rank percentile. Long, wide, red bars show increased significance. It was 20.2-fold upregulated in the sperm cells with abnormal morphology compared to controls. It was significantly downregulated in the PBMC from malaria infected patients compared to uninfected controls (fold = 4.5, q < 0.0005) and presymptomatic infected patients (fold = 3.4, q < 0.0005). It was also significantly downregulated in multiple independent cancer studies, including 13.3-fold downregulation in primary malignant melanoma samples, 3.6-fold downregulation in metastatic prostate cancer tumors and 3.4-fold downregulation in the plasma cells from patients with plasma cell leukemia.

Filtering revealed interesting connections between Nanog expression and disease state, infection and drug-response. Filtering by disease state comparison showed that Nanog was significantly upregulated in abnormal sperm morphology and significantly downregulated in malaria infection (Fig. 1, [15]). It was 20.2-fold upregulated in the sperm cells from male with consistent and severe teratozoospermia, a condition in which less than 4 percent of sperm cells are morphologically normal, compared to controls (q < 0.0005, rank = 0.5%ile, GDS2697). It was also significantly downregulated in the peripheral blood mononuclear cells (PBMC) from malaria infected patients compared to uninfected controls (fold = 4.5, rank = 3.3%ile, q < 0.0005) and presymptomatic infected patients (fold = 3.4, rank = 7.7%ile, q < 0.0005) (GDS2362). These studies suggest roles of Nanog in both abnormal sperm morphology and malaria infection. These findings have not been previously reported as only a few selected genes were described in the original publications [16,17], which is common for most microarray studies. Therefore, GeneChaser enabled the reuse of the whole data set of deposited microarray studies, instead of a few genes described in the publications.

As expected, Nanog was also significantly downregulated in various types of cancer and progression in human studies (Fig. 1, [15]). It was 13.3-fold downregulated in primary malignant melanoma samples (q < 0.0005) and 2.2-fold downregulated in benign skin nevi (q = 0.004) compared to normal skin samples (GDS1375). It was also significantly downregulated in metastatic prostate cancer tumors compared to benign tumors (fold = 3.6, q = 0.022) and clinically localized tumors (fold = 3.5, q = 0.024, GDS1439). It was 3.4-fold downregulated in the plasma cells from patients with plasma cell leukemia compared to patients with premalignant conditions (q = 0.017, GDS1067). The significant downregulation of Nanog in multiple independent studies of various types of cancer indicated a potential role in cancer and progression, consistent with previous reports linking cancer to stem cell-like phenotypes [18].

Filtering by agent comparisons showed that Nanog expression was significantly affected by certain drugs (Additional File 2, [19]). For example, it was 6.4-fold upregulated in non-small cell lung cancer after treatment with gemcitabine (q = 0.03, GDS2777). It was also 2.9-fold downregulated in PBMC of malaria infected patients after treatment with chloroquine (q = 0.003, GDS2362). Its expression in ES cells was downregulated after treatment with retinoic acid (RA), and upregulated after treatment of dimethyl sulfoxide (DMSO) (GDS1823). These drugs could potentially be used to regulate the expression of Nanog for functional study.

### Multiple gene search

*NANOG*, *OCT4(Pou5f1)*, *SOX2*, and *LIN28 *were previously found to be sufficient to reprogram human somatic cells to pluripotent stem cells that exhibit the essential characteristics of embryonic stem (ES) cells [14]. We performed a *multiple gene search *and found that they were all significantly differentially expressed in five mouse studies (q ≤ 0.05) (Additional File 3, [20]). The most significant change was that *Nanog *(fold = 1000, q = 0.006), *Oct4 *(fold = 333, q = 0.006), *Sox2 *(fold = 1000, q = 0.006) and *LIN28 *(fold = 143, q = 0.006) were all significantly upregulated in ES cells compared to hematopoietic stem cells (GDS2718). This confirmed their roles in ES cell development.

An age comparison showed that *Nanog *(fold = 2.5, q = 0.006), *Oct4 *(fold = 3.2, q = 0.036), *Sox2 *(fold = 9.7, q = 0.013) and *LIN28 *(fold = 4.5, q = 0.006) were all downregulated in the gonadal somatic cells from mouse embryo at 11.5 compared to 10.5 days post coitum (GDS1724), which spans the critical period of male sex determination. The significant downregulation of all four critical ES cell factors suggested a potential role of ES cell differentiation in sex determination, gonad differentiation, and sexual dysgenesis syndromes, which had not been described in the original publication [21].

A genotype/variation comparison showed that *Nanog *(fold = 1.14, q = 0.01), *Oct4 *(fold = 1.1, q = 0.029), *Sox2 *(fold = 1.1, q = 0.006) and *LIN28 *(fold = 1.1, q = 0.024) were all downregulated in stearoyl-CoA desaturase 1 (Scd1) deficient mice (GDS1517). Considering they were only 1.2-fold downregulated after the knockout of *Oct4 *in mice (GDS1824), these changes are still significant. This finding suggests that Scd1 might be an upstream regulator of these four ES cell factors.

These four genes were also significantly differentially expressed in 35 human studies (q ≤ 0.05) (Additional File 4, [22]). For example, *Nanog *(fold = 13.3, q < 0.0005), *Oct4 *(fold = 3.8, q < 0.0005), *Sox2 *(fold = 1.5, q = 0.018) and *LIN28 *(fold = 2.3, q = 0.033) were all significantly downregulated in primary malignant melanoma samples compared to normal skin samples (GDS1375).

**Figure 2:**
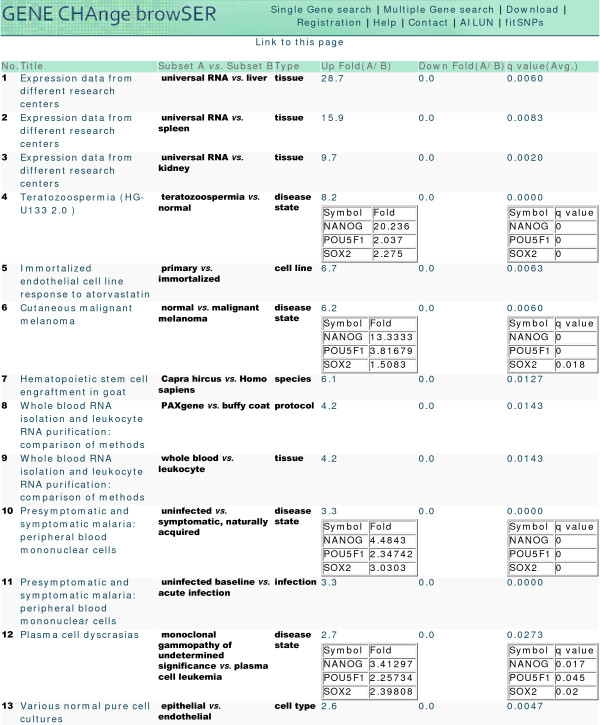
**Comparisons resulting differential expression of *Nanog*, *Oct4 *and *Sox2***. A *multiple gene search *for *Nanog, Oct4 (pou5f1)*, and *Sox2 *in human studies showed that they were all significantly upregulated in abnormal sperm morphology, and downregulated in malaria infection and cancer. *Nanog *(fold = 20.2, q < 0.0005), *Oct4 *(fold = 2.0, q < 0.0005) and *Sox2 *(fold = 2.3, q < 0.0005) were all significantly upregulated in the abnormal sperm morphology compared to controls. *Nanog *(fold = 4.5, q < 0.0005), *Oct4 *(fold = 2.3, q < 0.0005) and *Sox2 *(fold = 3.0, q < 0.0005) were all significantly downregulated in the PBMC from malaria infected patients compared to controls. They were also significantly downregulated in various types of cancers, such as plasma cell leukemia and malignant melanoma. Clicking the average q-values and fold changes opens values for individual genes.

To further investigate the role of ES cell factors in diseases, we performed another *multiple gene search *on *Nanog*, *Oct4 *and *Sox2 *and found that they were all significantly upregulated in abnormal sperm morphology, and significantly downregulated in malaria infection and cancer in human studies (Fig. 2, [23]). *Nanog *(fold = 4.5, q < 0.0005), *Oct4 *(fold = 2.3, q < 0.0005) and *Sox2 *(fold = 3.0, q < 0.0005) were all significantly downregulated in the PBMC from malaria infected patients compared to controls (GDS2362). The result indicates that key regulators of pluripotency are down-regulated in the response to malarial infection.

## Discussion

We developed GeneChaser to automatically analyze all GEO data sets (GDS), identify all experimental conditions in which a gene or set of genes was differentially expressed, and suggest biological functions, clinical implications, and regulators for genes of interest. We performed a *single gene search *for Nanog and found its most significant regulation is in embryonic stem (ES) cells compared to hematopoietic stem cells. This is consistent with our current understanding of Nanog in ES cell developments. We further found its potential association with abnormal sperm morphology, malaria infection and cancers. We also found four drugs that regulate the expression of Nanog, which could potentially be used for its functional studies.

We also showed how a *multiple gene search *for *Nanog*, *Oct4*, *Sox2 *and *LIN28 *confirms their most significant regulation in ES cells, and suggests their roles in the sex determination. We found that stearoyl-CoA desaturase 1 (Scd1) might be an upstream factor for all four ES factors. We further found that *Nanog*, *Oct4 *and *Sox2 *were all significantly upregulated in abnormal sperm morphology and dowregulated in malaria infection and cancer, which suggests a potential role of ES cell differentiation in these diseases.

Second generation microarray databases, such as GNF SymAtlas, Connectivity Map, L2L, Oncomine and Nextbio rely on manual curation of data. While these tools are extremely useful, they are also labor-intensive. With the advent of an automatic microarray annotation tool AILUN, GeneChaser analyzes all GEO data sets in a fully automatic way. This frees users from the limitations of manually curated data sets, and enables us to incorporate new data faster and more easily. In addition, in contrast to the small number of pre-established comparison types in the older databases, GeneChaser allows us to take advantage of all comparison types in GEO. As a result, we were able to perform complete group versus group comparisons of all GEO data sets, including numerous comparisons the original authors did not perform. These comparison types were also incorporated into GeneChaser's filters which enable users to quickly identify specific experimental conditions of interest. Finally, we designed color bars to represent output conditions that enable easy visualization and comparison according to different characteristics, including q-value, fold change, and rank percentile.

Of the second generation microarray databases, Nextbio contains gene profiling for a very large number of conditions. GeneChaser is different from Nextbio by offering a *multiple gene search *function, which we have shown here to be a powerful tool to identify conditions, such as the role of ES cell factors in sex determination, abnormal sperm morphology, malaria infection and cancer. GeneChaser also analyzed all 24 GEO-specified types of experimental conditions, while Nextbio only manually curated three types of experimental conditions. For example, a search of Nanog on Nextbio returned 210 conditions, while a *single gene search *of Nanog in GeneChaser returned 407 conditions (q ≤ 0.05). GeneChaser was also different from Nextbio by providing statistical comparisons with multi-test correction (q-value) and informative color bars to compare different conditions. Most importantly, the construction and update of GeneChaser are fully automatic, which enabled us to incorporate the data faster and easier. We are committing to refresh with the latest gene profiling database and update GeneChaser four times per year.

## Conclusion

In summary, GeneChaser analyzed all GEO data sets in an automated way to quickly deliver gene expression profiles under 12,658 experimental conditions across all species to the hands of biologists and clinicians without bioinformatics skills. We performed a *single gene search *for *Nanog *and a *multiple gene search *for *Nanog*, *Oct4*, *Sox2 *and *LIN28*, confirmed their roles in embryonic stem (ES) cell development, identified several drugs that regulate their expression, and suggested potential roles of ES cell factors in sex determination, abnormal sperm morphology, malaria infection, and cancer. With the expected doubling or tripling data accumulation in GEO, GeneChaser will quickly expand to a complete spectrum of biological and clinical conditions to elucidate biological functions, clinical implications, transcriptional regulators, and drug response for novel genes.

## Methods

### Microarray Data Analysis

Gene Expression Omnibus (GEO) is a centralized repository of gene expression data. Each array is recorded as a GEO sample (GSM). A group of related GSM from one study was linked together as a GEO series (GSE) according to the user submission. A collection of biologically and statistically comparable GSM from a study was then curated by GEO staff to form a GEO data set (GDS). All samples within a GDS were measured on the same platform with the same background processing and normalization, and their values were directly comparable. Some GSM were not included in the GDS. GEO staff categorized each GDS into subsets and annotated them with one of the 24 types.

We downloaded and parsed all SOFT files of GDS into a relational database using AILUN [8]. The types of expression values were recognized from "dataset_value_type" in the GDS SOFT files and transformed into log2 values as needed. We wrote an R script to perform all possible subset-versus-subset comparisons in each GEO-defined type in every GDS, ignoring subsets with less than 3 samples. For every comparison, we identified all differentially expressed probes with q-value ≤ 0.2 using the SAM function [10] in siggenes package of Bioconductor. We used AILUN [8] to translate differentially expressed probe IDs to Entrez Gene IDs. All q-values and fold changes were recorded. Because the number of differentially expressed genes varied among comparisons, a rank percentile was calculated for each gene based on its fold change among all changing genes (q ≤ 0.2) in each comparison.

In the original platform annotation files released from GEO, some probes were not annotated with any Entrez Gene ID, such as probe 575 in GPL5. An annotation of "FBgn0036403" was listed with a column header of "Platform_CLONEID". AILUN automatically recognized it as a FLYBASE ID and related it to an Entrez Gene ID 39556 using our universal gene identifier table, which contains 83 million identifiers for 3.6 million genes from 4578 species[8]. AILUN also removed all probes that were annotated with multiple Entrez Gene IDs to ensure a high-quality annotation.

We saved all performed comparisons in a table in MySQL database, which contains 12,658 comparisons. All differentially expressed genes along with their q values, fold changes, and rank percentiles were saved in a table, which contains 28 million entries. The whole process from data downloads, platform annotation, and gene identification, to database development was fully automated.

### Single gene search

GeneChaser directly retrieves qualified comparisons from our tables in MySQL. In *single gene search*, we first searched for a gene input in the official gene symbols in Entrez Gene and Homologene. If failed, we searched for it in our universal gene identifier table [8] to identify matching Entrez Gene IDs and Homologene group ID. When multiple Entrez Gene and Homologene IDs matched, they were all displayed with description for selection. Clicking an Entrez Gene ID returned comparisons where a gene was differentially expressed, while clicking a Homologene group ID returned comparisons where at least one gene in the group was differentially expressed.

Matched comparisons were displayed in color bars, which were created with the HTML *div *tag, with length = min (fold change, 10) × 50, width = ((1 - rank percentile) + 0.05) × 50 and color for q-value. Redder bars indicated better q-value. The fold change was calculated as the expression level change of subset A/B, which was listed under the color bar. When fold change was less than 1, we exchanged subset A and B to ensure that fold change was shown as larger than 1. Results were displayed as thresholds filtered by q-value, fold change, title keyword, and comparison types. Displayed graphic results could be saved as a high-quality image, which was created using PHP's GD library (bundled 2.0.28 compatible).

### Multiple gene search

Similar with *single gene search*, we first identified corresponding Entrez Gene IDs for two input gene lists. Because the majority of individual microarray studies measure gene expression in a single species, we restricted all input genes to be in the same species, which greatly reduced the number of matching Entrez Gene IDs. If multiple species or genes were matched, we displayed all with description for user selection.

We then retrieved all comparisons in which genes on the first list were upregulated and those on the second list were downregulated using user-defined q-value cutoffs. All comparisons were saved into a temporary table containing fold changes and q-values for both individual genes and the average change, which was then used to create a HTML table for display.

### User guide

GeneChaser is a gene-specific search tool across all experimental conditions in GEO. In *single gene search*, the input is a gene identifier, such as a gene symbol. For example, a search for "Nanog" returns a list of matching IDs from Entrez Gene and Homologene. Clicking an Entrez Gene ID returns experimental conditions in a single species, while clicking a Homologene ID returns experimental conditions in all species. For example, Nanog was measured in 5,706 comparisons, and differentially expressed in 217 (q ≤ 0.05, fold > 2) [13]. Each row in the output table lists Nanog expression in an identified comparison, and includes the rank of comparison, species, study title, a color bar representing the significance of the comparison, comparison type, fold change, rank percentile among all changing genes with q-value ≤ 0.2 in the comparison, and q-value. The color represents the q-value, with redder colors indicating higher significance. Bar length represents fold change with longer lengths for larger changes. Bar width represents rank percentile with greater widths for higher percentiles. Clicking on the title opens the original study with the raw GEO data. Clicking on the colored bar opens the original expression values for the probe in GEO, including present/absent calls for Affymetrix arrays. The two groups under comparison are listed under the color bar. For example, the first row indicates that Nanog was 1000-fold upregulated (q = 0.006, rank = 0.2%ile) in embryonic stem cell compared to hematopoietic stem cell in mice [13]. More detailed description is available by clicking *Legends *(top right).

Results can be threshold filtered by q-value, fold, rank percentile, and comparison types. Comparison types are defined by GEO. The comparison type filter is convenient for quickly identifying conditions of interest. For example, *disease state *lists all diseases where Nanog was differentially expressed. *Genotype/variation *and *strain *return mutation and knockout studies. *Agent *suggests drugs for regulating Nanog expression. Other frequently used comparison types include *infection*, *protocol*, *tissue*, *cell type*, *cell line*, *development*, and *age*. User can also use keywords to filter study titles. Results are initially sorted by q-values, but can be re-sorted by fold change, title, rank percentile, and species. Changes to filter thresholds and sort selections are retained if a user selects *Start Over *for a new search. Results can be displayed graphically or as text in *Display Results *(top left). Graphical results can be saved in a high quality PNG file using *Create Image*. Textual results can be downloaded as a tabbed text file for detailed analysis. A permanent link can be created by *Link to this page*, which can be forwarded to collaborators.

Similar to *single gene search*, *multiple gene search *takes a list of up- and downregulated genes as input and displays differentially expressed comparisons with average fold changes and q-values. Clicking the average values opens individual values for each input gene.

## Availability and requirements

**Project name**: GeneChaser

**Project homepage**: 

**Operating systems**: Platform independent

**Programming languages**: R and PHP

**Other requirements**: Web browser (supporting JavaScript)

**Any restrictions to use by non-academics**: License needed

## Abbreviations

ES cell: Embryonic Stem cell; GEO: Gene Expression Omnibus; GDS: GEO Data Set; GPL: GEO Platform; GSE: GEO Series; GSM: GEO sample; PBMC: Peripheral Blood Mononuclear Cells; SAM: Significance Analysis of Microarray.

## Authors' contributions

RC carried out microarray data parsing and database development, participated in the study design, microarray data analysis, and web server design, and drafted the manuscript. RM and AT participated in the web server design, and carried out web server development. SV participated in the manuscript editing and test cases. AB conceived of the study, and participated in the study design, microarray data analysis, web server design, and manuscript editing. All authors read and approved the final manuscript.

## Supplementary Material

Additional file 1 **Top conditions in which Nanog was significantly differentially expressed.** A single gene search result shows the top 12 biological and clinical conditions in which Nanog, or one of its orthologs, was differentially expressed (q ≤ 0.05, fold > 2). Click here for file

Additional file 2 **Agent comparisons showing differential expression of Nanog.** A single gene search result shows that Nanog, or one of its orthologs, was differentially expressed after drug treatments in four studies (q ≤ 0.05, fold > 2). Click here for file

Additional file 3 **Mouse studies showing differential expression of Nanog, Oct4, Sox2, and Lin28.** A multiple gene search result shows that Nanog, Oct4, Sox2, and Lin28 were differentially expressed in five mouse studies (q ≤ 0.05). Click here for file

Additional file 4 **Human studies showing differential expression of Nanog, Oct4, Sox2, and Lin28.** A multiple gene search result shows the top 14 human studies in which Nanog, Oct4, Sox2, and Lin28 were differentially expressed (q ≤ 0.05). Click here for file
