# Supplementary material for: GeneChaser: Identifying all biological and clinical conditions in which genes of interest are differentially expressed
Source: BMC Bioinformatics. 2008 Dec 18;9:548. doi: 10.1186/1471-2105-9-548 (PMC2629779; doi:10.1186/1471-2105-9-548)
Supplement: Additional file 1 — Top conditions in which Nanog was significantly differentially expressed. A single gene search result shows the top 12 biological and clinical conditions in which Nanog, or one of its orthologs, was differentially expressed (q ≤ 0.05, fold > 2). [file 1471-2105-9-548-S1.pdf]

GENE CHAnge browSER

Single Gene search | Multiple Gene search | Download | Registration | Help | Contact | AILUN | fitSNPs

Display Results : Graphical   Sort By Fold   Show 20   Filter with : Comparison type All   q value <=0.05   Fold >2   Rank All

Items 1 - 20 of 217 significant comparisons.   Page 1 of 11

HomoloGene group **78027** for NANOG,Nanog homeobox was differentially expressed in 217 of 5706 comparisons.

Create Image

Search by Title Keyword   Link to this page   Start Over   Legends

No.	Species Name	Title	Differential Expression (A *vs.* B)	Type	Fold (A/B)	Rank (%ile)	q value
1	Mus musculus	**Transcription factor Zfx deficiency effect on embryonic and hematopoietic stem cells**	embryonic stem cell ***vs.*** hematopoietic stem cell	cell type	1000.0	0.2	0.006
2	Mus musculus	**Preimplantation embryo development (MOE430A)**	blastocyst ***vs.*** oocyte	development stage	243.2	0.3	0.009
3	Mus musculus	**Preimplantation embryo development (MOE430A)**	8-cell ***vs.*** oocyte	development stage	138.1	0.5	0.009
4	Mus musculus	**Embryonic stem cell cholesterol metabolism mutants**	embryonic stem cell ***vs.*** liver	cell type	90.9	0.9	0.000
5	Mus musculus	**Preimplantation embryo development (MG-U74A)**	blastocyst ***vs.*** oocyte	development stage	87.5	1.5	0.012
6	Mus musculus	**Preimplantation embryo development (MOE430A)**	blastocyst ***vs.*** 2-cell	development stage	69.7	0.3	0.009
7	Homo sapiens	**Expression data from different research centers**	universal RNA ***vs.*** liver	tissue	62.5	0.2	0.006
8	Mus musculus	**Preimplantation embryo development (MOE430A)**	blastocyst ***vs.*** 1-cell	development stage	58.9	2.1	0.007
9	Mus musculus	**Preimplantation embryo development (MG-U74A)**	8-cell ***vs.*** oocyte	development stage	54.3	1.9	0.010
10	Mus musculus	**Preimplantation embryo development (MG-U74A)**	8-cell ***vs.*** 1-cell	development stage	52.9	4.1	0.014
11	Mus musculus	**Preimplantation embryo development (MG-U74A)**	blastocyst ***vs.*** 2-cell	development stage	45.4	0.8	0.047
12	Mus musculus	**Preimplantation embryo development (MOE430A)**	8-cell ***vs.*** 2-cell	development stage	39.6	0.2	0.011
