# Supplementary material for: GeneChaser: Identifying all biological and clinical conditions in which genes of interest are differentially expressed
Source: BMC Bioinformatics. 2008 Dec 18;9:548. doi: 10.1186/1471-2105-9-548 (PMC2629779; doi:10.1186/1471-2105-9-548)
Supplement: Additional file 2 — Agent comparisons showing differential expression of Nanog. A single gene search result shows that Nanog, or one of its orthologs, was differentially expressed after drug treatments in four studies (q ≤ 0.05, fold > 2). [file 1471-2105-9-548-S2.pdf]

GENE CHAnge browSER

Single Gene search | Multiple Gene search | Download | Registration | Help | Contact | AILUN | fitSNPs

Display Results : Graphical Sort By Fold Show 20 Filter with : Comparison type agent q value <=0.05 Fold >2 Rank All

Items 1 - 4 of 4 significant comparisons.

Page 1 of 1

HomoloGene group **78027** for NANOG,Nanog homeobox was differentially expressed in 4 of 5706 comparisons.

Create Image

Search by Title Keyword

Link to this page Start Over Legends

No.	Species Name	Title	Differential Expression (A *vs.* B)	Type	Fold (A/B)	Rank (%ile)	q value
			Fold 1 2 3 4 5 6 7 8 9				
1	Homo sapiens	**Bexarotene effect on gemcitabine resistant non-small lung cancer cell line**	gemcitabine ***vs.*** untreated	agent	6.4	1.6	0.030
2	Homo sapiens	**Bexarotene effect on gemcitabine resistant non-small lung cancer cell line**	vehicle ***vs.*** untreated	agent	4.6	7.6	0.043
3	Homo sapiens	**Presymptomatic and symptomatic malaria: peripheral blood mononuclear cells**	untreated ***vs.*** chloroguine treated	agent	2.9	3.5	0.003
4	Mus musculus	**Embryonic stem cell differentiation induced by various chemicals: time course**	RA ***vs.*** DMSO	agent	2.2	33.3	0.046
			Fold 1 2 3 4 5 6 7 8 9				

Display Results : Graphical Sort By Fold Show 20 Filter with : Comparison type agent q value <=0.05 Fold >2 Rank All

Copyright ©2008 All Rights Reserved. Comments & Questions to Butte Lab, **Webmaster** Last updated on 23rd May, 2008
