# Supplementary material for: GeneChaser: Identifying all biological and clinical conditions in which genes of interest are differentially expressed
Source: BMC Bioinformatics. 2008 Dec 18;9:548. doi: 10.1186/1471-2105-9-548 (PMC2629779; doi:10.1186/1471-2105-9-548)
Supplement: Additional file 3 — Mouse studies showing differential expression of Nanog, Oct4, Sox2, and Lin28. A multiple gene search result shows that Nanog, Oct4, Sox2, and Lin28 were differentially expressed in five mouse studies (q ≤ 0.05). [file 1471-2105-9-548-S3.pdf]

GENE CHAnge browSER

Link to this page

No.	Title	Subset A *vs.* Subset B	Type	Up Fold(A/B)	Down Fold(A/B)	q value(Avg.)
1	**Transcription factor Zfx deficiency effect on embryonic and hematopoietic stem cells**	embryonic stem cell *vs.* hematopoietic stem cell	cell type	**619.0**<br>Symbol / Fold<br>Lin28 / 142.857<br>Nanog / 1000<br>Pou5f1 / 333.333<br>Sox2 / 1000	**0.0**	**0.0060**<br>Symbol / q value<br>Lin28 / 0.006<br>Nanog / 0.006<br>Pou5f1 / 0.006<br>Sox2 / 0.006
2	**Sex specific transcription in somatic and reproductive tissues**	hypothalamus *vs.* liver	tissue	**19.1**	**0.0**	**0.0088**
3	**Gonadal somatic cells during the critical period of sex determination: time course**	10.5 dpc *vs.* 11.5 dpc	age	**5.0**<br>Symbol / Fold<br>Lin28 / 4.5045<br>Nanog / 2.48139<br>Pou5f1 / 3.20513<br>Sox2 / 9.70874	**0.0**	**0.0168**<br>Symbol / q value<br>Lin28 / 0.006<br>Nanog / 0.012<br>Pou5f1 / 0.036<br>Sox2 / 0.013
4	**Transcription factors Nanog and Oct4 knockdown effect on embryonic stem cells**	Control *vs.* Oct4 knockdown	protocol	**1.2**<br>Symbol / Fold<br>Lin28 / 1.11857<br>Nanog / 1.14416<br>Pou5f1 / 1.26422<br>Sox2 / 1.17647	**0.0**	**0.0105**<br>Symbol / q value<br>Lin28 / 0.016<br>Nanog / 0.011<br>Pou5f1 / 0.006<br>Sox2 / 0.009
5	**Stearoyl-CoA desaturase 1-deficient mutants on a very low-fat, high-carbohydrate diet: liver expression profile**	wild type *vs.* Scd1 null	genotype/variation	**1.1**<br>Symbol / Fold<br>Lin28 / 1.10988<br>Nanog / 1.13895<br>Pou5f1 / 1.09409<br>Sox2 / 1.10132	**0.0**	**0.0173**<br>Symbol / q value<br>Lin28 / 0.024<br>Nanog / 0.01<br>Pou5f1 / 0.029<br>Sox2 / 0.006

Copyright ©2008 All Rights Reserved. Comments & Questions to Butte Lab, **Webmaster** Last updated on 23rd May, 2008
