# Supplementary material for: GeneChaser: Identifying all biological and clinical conditions in which genes of interest are differentially expressed
Source: BMC Bioinformatics. 2008 Dec 18;9:548. doi: 10.1186/1471-2105-9-548 (PMC2629779; doi:10.1186/1471-2105-9-548)
Supplement: Additional file 4 — Human studies showing differential expression of Nanog, Oct4, Sox2, and Lin28. A multiple gene search result shows the top 14 human studies in which Nanog, Oct4, Sox2, and Lin28 were differentially expressed (q ≤ 0.05). [file 1471-2105-9-548-S4.pdf]

No.	Title	Subset A *vs.* Subset B	Type	Up Fold(A/B)	Down Fold(A/B)	q value(Avg.)
1	**Expression data from different research centers**	universal RNA ***vs.*** liver	tissue	**25.3**	**0.0**	**0.0060**
2	**Expression data from different research centers**	universal RNA ***vs.*** spleen	tissue	**15.1**	**0.0**	**0.0078**
3	**Expression data from different research centers**	universal RNA ***vs.*** kidney	tissue	**11.7**	**0.0**	**0.0015**
4	**Immortalized endothelial cell line response to atorvastatin**	primary ***vs.*** immortalized	cell line	**5.9**	**0.0**	**0.0053**
5	**Hematopoietic stem cell engraftment in goat**	Capra hircus ***vs.*** Homo sapiens	species	**5.4**	**0.0**	**0.0133**
6	**Cutaneous malignant melanoma**	normal ***vs.*** malignant melanoma	disease state	**5.2**<br>Symbol / Fold<br>LIN28 / 2.31481<br>NANOG / 13.3333<br>POU5F1 / 3.81679<br>SOX2 / 1.5083	**0.0**	**0.0127**<br>Symbol / q value<br>LIN28 / 0.033<br>NANOG / 0<br>POU5F1 / 0<br>SOX2 / 0.018
7	**Glioma-derived stem cell factor effect on angiogenesis in the brain**	oligodendrogliomas ***vs.*** non-tumor	disease state	**2.1**	**0.0**	**0.0078**
8	**Glioma-derived stem cell factor effect on angiogenesis in the brain**	tumor grade II ***vs.*** not applicable	tissue	**2.1**	**0.0**	**0.0068**
9	**Carcinoma in situ lesions of the urinary bladder**	normal urothelium ***vs.*** superficial transitional cell carcinoma w/ surrounding carcinoma in situ lesion	specimen	**1.9**	**0.0**	**0.0005**
10	**Carcinoma in situ lesions of the urinary bladder**	carcinoma in situ lesion ***vs.*** superficial transitional cell carcinoma w/ surrounding carcinoma in situ lesion	specimen	**1.9**	**0.0**	**0.0110**
11	**Carcinoma in situ lesions of the urinary bladder**	normal urothelium ***vs.*** muscle invasive carcinoma	specimen	**1.7**	**0.0**	**0.0010**
12	**Carcinoma in situ lesions of the urinary bladder**	carcinoma in situ lesion ***vs.*** muscle invasive carcinoma	specimen	**1.7**	**0.0**	**0.0105**
13	**Transmigrated neutrophils in the alveolar space of endotoxin-exposed lung**	70 ***vs.*** 32	individual	**1.6**	**0.0**	**0.0413**
14	**Transmigrated neutrophils in the alveolar space of endotoxin-exposed lung**	70 ***vs.*** 48	individual	**1.5**	**0.0**	**0.0357**
